# Is the Brain Undernourished in Alzheimer’s Disease?

**DOI:** 10.3390/nu14091872

**Published:** 2022-04-29

**Authors:** Roberto Aquilani, Alfredo Costa, Roberto Maestri, Matteo Cotta Ramusino, Giulia Perini, Mirella Boselli, Paolo Iadarola, Daniela Buonocore, Manuela Verri, Maurizia Dossena, Federica Boschi

**Affiliations:** 1 Department of Biology and Biotechnology “Lazzaro Spallanzani”, University of Pavia, 27100 Pavia, Italy; dottore.aquilani@gmail.com (R.A.); piadarol@unipv.it (P.I.); daniela.buonocore@unipv.it (D.B.); manuela.verri@unipv.it (M.V.); maurizia.dossena@unipv.it (M.D.); 2 Unit of Behavioral Neurology and Center for Cognitive Disorders and Dementia, IRCCS C. Mondino Foundation, 27100 Pavia, Italy; alfredo.costa@mondino.it (A.C.); matteo.cottaramusino@mondino.it (M.C.R.); giulia.perini@mondino.it (G.P.); 3 Department of Brain and Behavioral Sciences, University of Pavia, 27100 Pavia, Italy; 4 Department of Biomedical Engineering of the Montescano Institute, Istituti Clinici Scientifici Maugeri IRCCS, 27040 Montescano, Italy; roberto.maestri@icsmaugeri.it; 5 Unità di Riabilitazione Neuromotoria Gravi Cerebrolesioni Acquisite, Istituti Clinici Scientifici Maugeri Spa Società Benefit, IRCCS, 27040 Montescano, Italy; mirella.boselli@icsmaugeri.it; 6 Department of Drug Sciences, University of Pavia, 27100 Pavia, Italy

**Keywords:** Alzheimer, CSF amino acid levels, amino acid CSF/plasma ratio, nutritional state

## Abstract

Cerebrospinal fluid (CSF) amino acid (AA) levels and CSF/plasma AA ratios in Alzheimer Disease (AD) in relation to nutritional state are not known. Methods: In 30 fasting patients with AD (46% males, 74.4 ± 8.2 years; 3.4 ± 3.2 years from diagnosis) and nine control (CTRL) matched subjects, CSF and venous blood samples were drawn for AA measurements. Patients were stratified according to nutritional state (Mini Nutritional Assessment, MNA, scores). Results: Total CSF/plasma AA ratios were lower in the AD subpopulations than in NON-AD (*p* < 0.003 to 0.017. In combined malnourished (16.7%; MNA < 17) and at risk for malnutrition (36.6%, MNA 17–24) groups (CG), compared to CTRL, all essential amino acids (EAAs) and 30% of non-EAAs were lower (*p* < 0.018 to 0.0001), whereas in normo-nourished ADs (46.7%, MNA > 24) the CSF levels of 10% of EAAs and 25% of NON-EAAs were decreased (*p* < 0.05 to 0.00021). CG compared to normo-nourished ADs, had lower CSF aspartic acid, glutamic acid and Branched-Chain AA levels (all, *p* < 0.05 to 0.003). CSF/plasma AA ratios were <1 in NON-AD but even lower in the AD population. Conclusions: Compared to CTRL, ADs had decreased CSF AA Levels and CSF/plasma AA ratios, the degree of which depended on nutritional state.

## 1. Introduction

There is currently no treatment able to stop the progression of Alzheimer’s disease dementia [1]. It is desirable that a future improved understanding of brain metabolic alterations in Alzheimer’s disease (AD) might provide the possibility of non-pharmacological interventions to prevent or slowdown the progression of this disorder.

Patients with AD (hereinafter ADs) have very complex brain metabolic alterations, including insulin and insulin growth factor 1 (IGF-1) resistances [2,3], reduced glucose transporters (Glut 1, Glut 3) [4], abnormal glycolysis pathway [5,6,7,8], oxidative stress [9,10,11,12] mitochondrial dysfunction [13,14] and altered mitochondrial enzyme activities [15,16,17,18,19,20], and disruption of Ca^2+^ homeostasis [21], all of which are factors leading to reduced brain glucose breakdown and utilization (Cerebral glucose hypometabolism: CGH), responsible for low energy generation (ATP). CGH precedes the onset symptoms of AD [22,23,24,25].

Lack of energy and oxidative stress, indeed, initiate and worsen over time the synaptic pathology in AD [12,14,26,27], as adequate energy availability is essential for normal synaptic activity [12]. Hyperoxidation of lipid, protein, nuclear and mitochondrial DNA is associated with plaques and tangles leading to synaptic dysfunction and disruption [12].

Abnormal mitochondrial enzyme activities are associated with cognitive impairment [28] and clinical disability [16]. Accumulations in mitochondria of Amyloid Beta (Aβ) and Amyloid Precursor Peptide (APP) make mitochondria dysfunctional [29].

In the context of CGH, the brain should rely on alternative substrates to generate energy, such as Amino Acids (AAs), lactate, pyruvate, and ketone bodies [30,31], given that it cannot rely on free fat acid oxidation due to the lack of enzymes responsible for β-oxidation in neurons [31]. With respect to AAs in the early onset of AD and experimentally induced hypoglycemia, elevated brain utilization of these substrates for energy generation has been indirectly documented through a large cerebral release of AAs and ammonia [32,33,34,35]. Elevated brain utilization of AAs could explain their reduced levels in the cerebrospinal fluid (CSF) of these subjects [36].

To the best of our knowledge, a comprehensive study on all standard individual and collective CSF AAs in their mutual interrelationships with the corresponding plasma levels, the metabolic development of AD markers and the patients’ nutritional state has not so far been carried out. We believe that the findings from such a type of study might prove useful for future investigations addressing the possibility of exogenously manipulating the brain’s amino acid metabolism.

In the current prospective, observational study, we hypothesized that patients with advanced AD, compared to non-AD subjects (NON-ADs), may have lower CSF AA levels and lower CSF/plasma AA ratios, suggesting brain overutilization of AAs. The rationale of our hypothesis is based on the following two arguments: first, the CSF compartment is in free communication with the interstitial fluid (ISF) surrounding neurons and glia [37] and drains the brain ISF [38], in this way removing the products of brain metabolism [39,40]. Second, in turn, the composition of CSF influences the composition of brain ISF and the neuronal function [37,41], reflecting the biochemical alterations of the brain in AD [40,42]. 

The second hypothesis we formulated was that the nutritional state of ADs could influence the AA levels in the CSF compartment and the CSF/plasma ratios, as circulating AAs, which normally enter the brain in mutual competition [43,44,45], may be reduced in malnourished ADs [46]. Following our reasoning, malnourished ADs compared to normo-nourished ones would exhibit even lower CSF AA levels and CSF/plasma ratios. Therefore, the study focused on determination of the levels of AAs in the CSF compartment and the CSF/plasma AA ratios in AD patients stratified according to nutritional state.

## 2. Methods

This prospective observational study included parts of the AD dementia and CTRL patient populations investigated by our team in a previous study [46], in whom cerebrospinal fluid (CSF) had been drawn from the lumbar tract. Summarized below are the subjects’ main characteristics and the procedures they underwent.

The patients were cognitively impaired (Mini Mental State Examination, MMSE, 18.4 ± 7.1 scores) and underwent diagnostic testing in the Department of General Neurology at the National Institute of Neurology IRCCS Mondino Foundation, Pavia, Italy, between 2014 and 2018.

Clinical diagnosis of dementia was formulated following the current research criteria for Alzheimer’s disease [47]. CTRL were selected among patients hospitalized in the same department for reasons unrelated to cognitive disorders or inflammatory diseases. The study was approved by the local Ethical Committee at the time of the previous study [47] (Project identification code: MAIR 2016; ethical approval: 130/INT/2016; date: 8 September 2016).

All procedures complied with the ethical standards of human experimentation and with the Helsinki Declaration of 1975, as revised in 2008. The study was approved by the local Ethics Committee (Project identification code: MAIR2016). Participants or their legal representatives provided written informed consent for participation in the study. No participant received financial compensation.

### 2.1. Procedures

#### 2.1.1. Clinical Evaluation

All enrolled patients underwent complete clinical, neurological, and neuropsychological assessment and brain magnetic resonance imaging (MRI). 

In addition to physical examination, routine blood tests, anthropometric measurements (body weight, BW, kg; height, cm; body mass index, BMI, kg/m^2^; Mid-Arm Circumference, MAC, cm), clinical evaluation and assessment of patients’ nutritional state by means of Mini Nutritional Assessment (MNA) score [48] were carried out. MNA score < 17 denotes a state of malnutrition, a score between 17 and 24 denotes risk of malnutrition, and a score > 24 identifies a normal nutritional state.

#### 2.1.2. Plasma and CSF AA Measurements

In both ADs and CTRL, at 8 a.m. after 12 h of overnight fasting, blood samples were drawn from an antecubital vein and immediately delivered to the laboratory, where plasma was obtained from heparinized blood using centrifugation (800× *g*, 15 min) in order to measure plasma AA levels. At the same time, 2 mL of CFS samples were drawn from the lumbar tract and immediately delivered to the laboratory.

Using CSF and plasma AA levels the CSF/plasma AA ratios were calculated.

#### 2.1.3. Assessment of AA Concentrations

The determination of AA concentrations, both in CSF and plasma, was achieved following the procedure described elsewhere [49]. AA concentrations were expressed in µmol/L. We measured all essential AAs (EAAs): lysine, threonine, phenylalanine, methionine, and tryptophan; the Branched-Chain AAs (BCAAs): leucine, valine, and isoleucine; and all non-essential AAs (NEAAs): aspartic acid, glutamic acid, histidine, asparagine, serine, glutamine, arginine, citrulline, glycine, alanine, tyrosine, and ornithine. 

The Tryptophan Ratio (Trp ratio) was calculated as follows:

trypthophan/(BCAA + phenylalanine + tyrosine)

#### 2.1.4. Insulin and HOMA-IR

Among the blood variables, plasma insulin levels (µU/mL) were measured using the Cord-CT Radioimmunoassay Kit CIS (France) and Coat A Count Insulin (D.P.C., Los Angeles, CA, USA) commercial kits. Insulin resistance was calculated using the Homeostasis Model assessment (HOMA; normal value < 2.4) [50,51].

### 2.2. Objectives

The first objective of the study was to document possible differences between ADs and CTRL in CSF AA levels and CSF/plasma ratio.

The second objective was to document whether there were differences in CSF AA levels and CSF/plasma ratio related to AD nutritional state.

### 2.3. Statistical Analysis

The normality of all variables was assessed using the Shapiro–Wilk statistical test, supported by visual inspection. Since several variables violated the assumption of normality, central tendency and dispersion of continuous variables were reported as mean ± SD, but nonparametric tests were used. Accordingly, between-groups comparisons were carried out using the Mann–Whitney U-test (for two groups) or the Kruskal–Wallis test (for three or more groups). A significant result on the Kruskal–Wallis test was followed by post-hoc analysis (Dunn–Sidak) to compare pairs of groups. 

Descriptive statistics for discrete variables were reported as percentage frequency. 

Associations between variables were assessed via both Pearson correlation coefficient and Spearman rank correlation coefficient. 

All statistical tests were two-tailed and statistical significance was set at *p* < 0.05. All analyses were carried out using the SAS/STAT statistical package, release 9.4 (SAS Institute Inc., Cary, NC, USA).

## 3. Results

### 3.1. Patients’ Clinical Characteristics

It can be seen from the data in Table 1 that AD patients, as an overall group, had normal body weight (BMI) and biohumoral variables, but presented with a state of light inflammation (ESR) and insulin resistance (HOMA-IR). According to MNA criteria, 16.7% of patients (n = 5) were malnourished (group 1), 36.6% (n = 11) were at risk for malnutrition (group 2), and 46.7% (n = 14) were normonourished (group 3).

### 3.2. CSF and Plasma AA Levels in ADs after Stratification by Nutritional Status

Table 2 and Table 3 show the CFS AA levels and plasma AA levels in ADs and in CTRL, respectively. Compared with CTRL, levels of AAs in CSF and plasma in ADs were reduced by 75% and 31.6%, respectively. However, in the CSF compartment, the numbers and types of AAs reduced varied in relation to patient nutritional state (Table 4). Given that Group 1 (malnutrition) and Group 2 (at risk) had similar numbers and types of altered CSF AA (with the exception of alanine, present only in group 2, and aspartic acid, present only in group 1), these groups were pooled together to form a combined group (CG) (named the “nutritionally-deteriorated” group). In CG, the alterations in numbers and types of AAs were wider than in Group 3 (normal nutrition). For clarity, these results were synthesized in Table 5 and Table 6.

Compared to CTRL, CG had lower plasma levels of glutamate, alanine, isoleucine, leucine, total AAs (TAAs), and Essential AAs (EAAs), and a lower EAA/TAA ratio (Table 7). Compared to CTRL, group 3 (normonutrition) had lower plasma levels of alanine and higher plasma BCAA/EAA and BCAA/TAA ratios. Plasma histidine levels were higher in each AD subgroup compared to CTRL.

In the CSF compartment (Table 8), compared to CTRL, CG and group 3 had in common significant reductions in levels of asparagine, serine, glutamine, glycine, arginine, tryptophane, phenylalanine, Trp ratio, TAAs, indicating that these altered AAs were independent of nutritional state. In addition, the CG had reduced levels of threonine, alanine, valine, methionine, isoleucine, leucine, lysine, EAAs, and BCAAs. Thus, deterioration of nutritional state in ADs was associated with wider reductions in AAs. In the CG, leucine, isoleucine, threonine, TAAs, and EAAs were reduced both in plasma and CSF.

Table 9 shows the CSF/plasma AA ratios in CTRL and ADs as an entire population. In CTRL the ratios were <1, indicating that the AA levels in CSF were lower than those in plasma, with the exception of glutamine and to a lesser extent BCAA/EAA whose CSF/plasma ratios were >1. Compared to CTRL, ADs had significantly lower CSF/plasma AA ratios, including glutamine and BCAA/EAA.

Table 10 shows that CSF/plasma AA ratios were similar among the normonourished and nutritionally deteriorated patients.

To sum up, compared to CTRL, ADs had important reductions in numbers and types of CSF AAs. Moreover, patients’ stratification according to nutritional state indicated the existence of two CSF AA profiles: one in which reduced AAs were independent of nutritional state, the other with a further reduction in AAs associated with a deteriorated nutritional state. Moreover, in CTRL subjects the CSF/plasma AA ratios were <1 but >1 for glutamine and the BCAA/EAA ratio. In ADs, CSF/plasma ratios were even lower than in CTRL, including glutamine and the BCAA/EAA ratio.

### 3.3. Correlations

Table 11 shows the correlations between plasma insulin levels, CSF AA levels, and markers of AD pathology.

**Figure 1:**
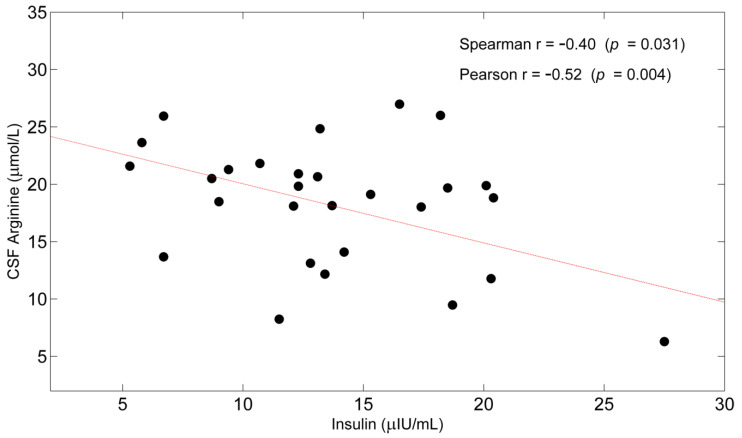
Scatterplot of Cerebrospinal fluid (CSF) Arginine as a function of Insulin. Spearman’s r and Pearson’s r are also reported with corresponding *p*-values.

Insulinemia was inversely correlated with CSF arginine (r = −0.52; *p* = 0.004) (Figure 1), whereas no other relationship was found with other CSF AAs, nor with Aβ, tau, or phosphor tau levels.

**Figure 2:**
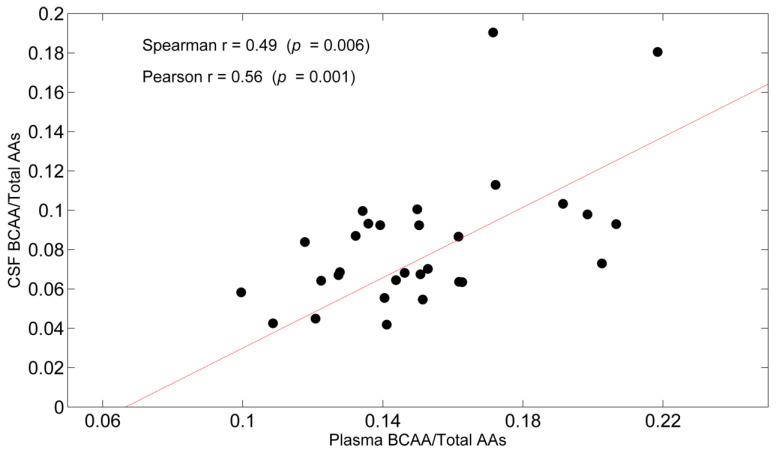
Scatterplot of Cerebrospinal fluid (CSF) Branched-Chain Amino Acids (BCAA)/Total Amino Acids (Total AAs) as a function of Plasma BCAA/Total AAs. Spearman’s r and Pearson’s r are also reported with corresponding *p*-values.

Correlations between CSF AAs and plasma AAs were evidenced both in ADs (Table 12) and CTRL (Table 13). In AD, an inverse correlation in glutamine levels was observed between plasma and CSF, whereas all BCAAs, the BCAA/TAA ratio (Figure 2) EAA/Total AA, and BCAA/EAA were positively associated.

In CTRL, the study found positive links for serine (r = + 0.96; *p* = 0.0007), BCAA/TAA ratio (r = + 0.90; *p* = 0.0005), and valine (r = + 0.74; *p* = 0.046).

## 4. Discussion

The study confirms the initial hypothesis that ADs, compared to controls, may have reduced CSF AA levels and lower CSF/plasma AA ratios and that the amount of CSF AA reduction is in part dependent on patients’ nutritional state.

The patients’ stratification by nutritional state identified two patterns of reduced CSF AA levels, one that was independent of nutritional state (found therefore in all ADs), and another that was found in the nutritionally deteriorated group only (i.e., group 1 + group 2). The latter group of ADs therefore exhibited both nutritionally independent and nutritionally dependent CSF AA reductions.

The lower CSF/plasma ratios in ADs, both as an entire group and after stratification by nutritional state, suggest that AA reductions in CSF may be due not only to insufficient supply of AAs to the brain from plasma but also to brain overutilization. The following factors may sustain the hypothesis of brain AA overutilization in ADs. First, as shown in the current study, 75% of the AAs in CSF but only 31.6% of the AAs in plasma were reduced. Second, the CSF levels of several AAs, including two EAAs, were lower in normonourished ADs despite their normal plasma levels. Third, as previously documented, AA/protein dysmetabolism is more severe in the AD brain than in extracerebral areas [52]. Another investigation reported the presence of increased urea levels in CSF [52,53,54].

For more clarity, brain AA alterations will be discussed below as derived from two main potential mechanisms, one of which may be refereed to as “general” and the other “specific”, while being aware that these two are intimately and inextricably interconnected.

### 4.1. Some Potential General Mechanisms for Reduced CSF AA

In addition to the need for energy production [31,55], insulin resistance, the synthesis of neuropathology markers of AD, and the mutual influence between insulin resistance and biosynthesis of AD markers are among the mechanisms able to prime/enhance brain AA overutilization.

The development of insulin resistance [2,3,56] might be favored and/or heightened by gut dysbiosis [57,58] that, via intestinal translocations of bacteria, bacterial amyloids and toxins, may increase cerebral inflammation brought about by Aβ [59]. Insulin resistance/hyperinsulinemia further reduces the residual utilization of glucose in mitochondria by blocking the mitochondrial pyruvate dehydrogenase enzyme complex [60].

Aβ itself can cause increased brain AA utilization, as demonstrated in both AD patients [61] and in AD model mice [62], via activation of mTOR signaling. On one hand, mTOR increases tau protein synthesis, the main component of the neurofibrillary tangles, [63] and, on the other hand, it stimulates mitochondrial activity [64]. In turn, activated mTOR can increase AA consumption for the synthesis of Aβ and tau protein [61]. Supporting the anabolic relationship between Aβ and mTOR, the inhibition of mTOR in AD model mice can reduce Aβ and tau protein levels, leading to the recovery of cognitive function [65,66]. Of note, BBB alteration itself contributes to increased brain AA utilization by upregulating Amyloid Precursor Protein (APP) expression and Aβ deposition [67]. 

The interrelationship between altered brain insulin signaling and the biosynthesis of AD markers may be another mechanism leading to brain AA overutilization. Hyperinsulinemia indeed increases tau phosphorylation [68,69], favors the formation of senile plaques [70] and inhibits degradation of extracellular neuronal Aβ by blocking the insulin-degrading enzyme [71]. In turn, both Aβ and tau worsen brain insulin signaling [72], in particular in synaptic areas [73]. A puzzling problem might be the coexistence of insulin resistance/hyperinsulinemia with reduced insulin receptor density in brain structures (by up to 80% in severe AD) [74,75]. Probably, chronic hyperinsulinemia/cerebral insulin resistance may downregulate and decrease the affinity of brain insulin receptors [74,76]. In the current study, we did not find significant correlations between insulinemia/IR and CSF Aβ or tau levels. A possible explanation is that this association might be more easily found within the brain structures. 

It is likely that low CSF AA levels were not due to the blood–brain barrier (BBB)-restricted entry of AAs into the brain from plasma, or due to Choroid Plexuses (CPs) removing AAs from CSF and/or reducing AA secretion into CSF, which are both mechanisms that, under physiological conditions, determine brain AA availability [41]. BBB and CP activities, indeed, are altered in AD. Early BBB breakdown and dysfunction occur before the development of dementia and brain atrophy [77,78,79,80], as also confirmed in post-mortem studies [81]. In AD, CPs undergo epithelial atrophy, basement membrane thickening, and stroma fibrosis [39]. Therefore, it is most likely that the reductions in CSF AA levels may be due to increased brain uptake [41] and consumption of AAs.

In AD, elevated urea cycle activity and elevated urea levels, both in the TCA cycle [52] and in CSF (not measured in the present study) [52,53,54], support brain AA overutilization. An excess of brain AA utilization has a negative impact on brain function as, in addition to increased urea levels, causes hyperammonia, given that neurons and glia lack all the enzymes of urea cycle [82]. Hyperammonia worsens brain dysfunction, as it is implicated in several metabolic processes including, to name only a few, oxidative damage [83,84] impaired mitochondrial activities [85,86] (also in synaptic regions [87]), and decreased astrocyte glutamine formation via reducing glutamine synthetase enzyme to scavenge ammonia [88]. Urea itself, if elevated, is neurotoxic [89]. Within the brain, urea is also produced from transaminase activities in neurons and astrocytes, involving alanine, aspartic acid, glutamate [52]. 

**Figure 3:**
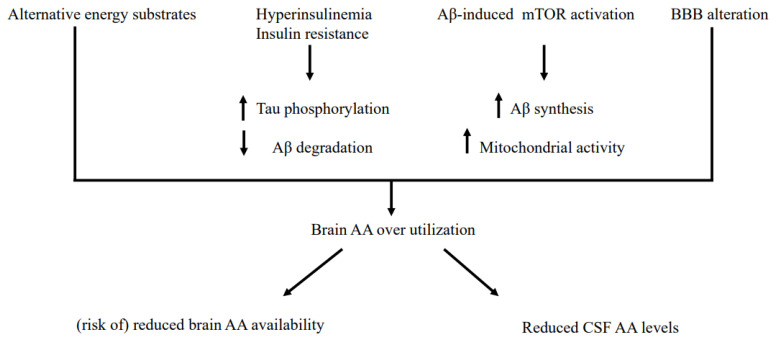
Some general potential mechanisms underlying brain amino acid overutilization and reduced CSF amino acid levels. 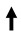 increase 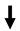 decrease. BBB: Blood-Brain-Barrier.

Some potential mechanisms underlying reduced CSF AA levels are summarized in Figure 3.

In summary, increased AA utilization in the AD brain might over time reduce the balance between positive effects (energy production) and negative ones (hyperammonia, high urea levels, increased synthesis of AD markers), resulting in abnormal neuron and glia death [31].

### 4.2. Some Potential, Specific Mechanisms for Reduced CSF AA Levels Independently of Patients’ Nutritional Status

The reductions in CSF asparagine, serine, glutamine, glycine, arginine, tryptophan, phenylalanine and Trp ratio were independent of patient nutritional state, suggesting that they were linked to AD per se; the brain may also use these AAs for specific metabolic tasks.

Low CSF serine and glycine confirm the findings from a post-mortem study [52] documenting the decrease in these AAs in the hippocampus, entorhinal cortex, middle-temporal gyrus, sensory cortex, motor cortex, cingulate gyrus, and cerebellum. Among the factors responsible for their reduction, increased utilization in the glycolytic pathway to generate energy, to increase/maintain glutathione system [90] and for neurological functions might play a major role. In the brain, serine can be converted into the stereoisomer D-serine, which is a potent activator of the NMDA glutamate receptor, which in turn is involved in the formation of new synapses and essential for learning and memory processes [46,91,92]. The importance of serine for brain activity may be inferred by the very strong correlation found in this study between plasma and CSF serine levels. Impaired serine production in astrocytes contributes to cognitive dysfunction in AD [92].

Glycine is normally produced by the brain [40]. Its reduction [36] may be due to both reduced formation from its immediate precursor, serine, and increased utilization in the glutathione system pathways. Low brain glycine may contribute to synaptic and brain toxicity, as this AA functions in the brain as a inhibitory neurotransmitter, similarly to GABA, which may be reduced in AD CSF [42].

Low asparagine could be due to reduced conversion of oxalacetate [93] from an altered TCA cycle. Its reduction may negatively impact cell proliferation and the urea cycle, given that this AA is a powerful stimulator of ornithine decarboxylase and cell proliferation [94].

Low CSF glutamine levels were in line with some studies [95,96] and contrasted with a recent investigation [97]. Glutamine is normally produced by brain [40] and its reduction is not due to lack of glutamate (this being similar between AD and CTRL) but instead may derive from both increased utilization for energy production [96] in the mitochondrial TCA cycle [98] and reduced synthesis due to impaired activity of the oxidized glutamine synthase enzyme (GS) [9,99,100]. Of note, the compromising of GS activity occurs early in AD [101]. Low brain glutamine in astrocytes reduces the capacity for detoxifying ammonia and leads to reduced Aβ autophagy capacity [101,102,103]. Under normal conditions, glutamine is neuroprotective [101]. Supporting this, CSF glutamine and glutamate are inversely correlated with the amyloid tau index [97], and GS is negatively associated with t-tau and p-tau [104]. Glutamine CSF/plasma ratio was >1 in CTRL, indicating the importance of brain production of this amino acid under normal conditions. This suggests that glutamine is exported from the brain rather than imported into the brain from plasma.

Low CSF arginine [42] may derive from its overconsumption in increased urea cycle activity, as well as in the enzymatic activities of nitric oxide synthase (NOS) and arginase where arginine serves as substrate. NOS in AD is upregulated [105] and elevated production of NO is responsible for increased nitrosative stress [106], causing mitochondrial dysfunction and the degeneration of both synapses and neurons [107]. Another arginine-consuming reaction is the production of the inhibitory neurotransmitter GABA through its conversion to ornithine [108]. Low arginine leads to disruption of the urea cycle, and consequently to hyper-ammonia, endothelial dysfunction and reduced synthesis of creatine and creatine kinase BB, which results in worsening neuronal energy deficits.

In the case of AD, the only CSF amino acid that significantly correlated with insulinemia was arginine. This correlation may be explained by the hyperinsulinemia-induced upregulation of brain arginase activity, as observed in obesity [109]. 

Increased arginase activity consumes arginine and reduces NO synthesis, as arginase competes with NOS for the same arginine substrate. In brief, whatever the prevalent mechanisms, the upregulation of the two enzymes (NOS and arginase) competing for the same substrate (arginine) greatly contributes to reducing brain arginine availability.

Two Essential Amino Acids (EAAs), tryptophan and phenylalanine, proved to be decreased in AD CSF. Being precursors of the neurotransmitter serotonin and catecholamines dopamine, norepinephrine, epinephrine, and tyramine, their reduction suggests that brain deficits in neurochemical transmission contribute to synapse dysfunction and the loss of synapses, neurons and neuronal networks [50]. 

The alterations in chemical neurotransmitters occur early in AD, before the pathological markers of AD or the loss of neurons [50].

The decrease in tryptophan may be attributed to activation of the kynurenine pathway [110], which is upregulated in AD [111,112]. The diversion of tryptophan towards the kynurenine pathways reduces serotonin formation, contributing to AD pathogenesis. Another potential mechanism for reduced tryptophan may be its increased utilization for brain protein synthesis, given the important role played by this amino acid in protein synthesis [50]. The loss of serotoninergic neurons correlates with AD severity and learning and memory impairments [50].

Previous investigations reported that phenylalanine was increased in the brain [52] and decreased in serum [113] of AD patients. These findings are the opposite of the results of the current investigation. An explanation for these contradictory results may be that the previous studies were carried out on two different AD populations and using two different methodologies, one of which involved examination of post-mortem patient brains [52]. The measurements of phenylalanine levels in plasma and CSF in the present study were concomitantly carried out on a unique AD group. This explanation is also valid for tryptophan, which, in the above studies, was decreased in serum and increased in the brain.

Low phenylalanine levels may result from its use for protein synthesis [50], increased breakdown in the TCA cycle for energy production, and residual catecholamine formation [114]. The depletion of catecholamine neurotransmitters impairs behavioral and cognitive functioning, in particular under stressful conditions and during aging [1]. Impaired dopamine neurotransmission causes low performance in the spatial working memory and spatial recognition memory [115,116]. Moreover, reduced brain catecholamine availability increases patient perception of fatigue [117].

Low Trp ratio suggests a greater prevalence of the deficit in serotoninergic transmission compared to the catecholaminergic deficit. 

### 4.3. Some Potential, Specific Mechanisms Underlying Reduced CSF Levels in Nutritionally-Deteriorated AD

In nutritionally deteriorated ADs, CSF levels of six EAAs (the three BCAAs plus threonine, methionine, lysine), of alanine, and of TAAs and EAAs as a group were reduced. Leucine, isoleucine, alanine, TAAs, and EAAs were altered both in plasma and CSF. Thus, the brain availability of these AAs, in particular the essential leucine and isoleucine, was in part conditioned by their limited supply in plasma [118,119], and hence by abnormalities of metabolism in extracerebral areas [46]. By contrast, brain reductions in threonine, lysine, and valine would be due to their brain overutilization (plasma methionine was not available; thus, the level of this AA can be discussed only in terms of brain content).

Chronic protein breakdown [46] and/or insufficient dietary BCAA intake may explain the lack of leucine and isoleucine in both plasma and CSF. The importance of circulating BCAA for brain BCAA availability is highlighted by the correlations between plasma and CSF BCAA found in the current investigation, both in CTRL and in ADs. The strong correlations of the BCAA/TAAs ratio and valine between plasma and CSF both in controls and in ADs indicate that the higher the plasma levels of BCAAs among the circulating AAs, the higher their contribution to AA levels in the CSF compartment.

The differences in degree in the above correlations between ADs and controls for BCAA/TAAs ratio and valine might confirm that, in AD, an important role may be played by brain overconsumption of BCAA for both protein synthesis and (in particular for valine) for TCA cycle activity for energy generation. 

Brain BCAA overutilization could occur to facilitate the synthesis of phosphorylated tau protein, the main component of neurofibrillary tangles, due to increases in the process of mTOR autophosphorylation [61]. Increased mTOR activation may be induced by Aβ levels [63] and by BCAA themselves, mainly leucine [120], which are potent activators of mTOR signaling for protein synthesis.

The reductions in leucine and valine in CSF confirm the findings from a previous study [36], which also documented a correlation between plasma and CSF valine levels and memory and cognitive functions.

Based on the background of existing literature, it is unclear whether BCAA may potentially exert neuroprotective or toxic effects.

Some experimental investigations reported unfavorable effects on the development and progression of AD [121,122]. In the AD mouse model, tau hyperphosphorylation was decreased by a protein-restricted diet [123]. In contrast, clinical studies reported favorable effects on the retrieval of neurocognitive and neuropsychological functions after BCAA supplementation in patients with cirrhosis and chronic hepatic encephalopathy [124,125], with latent portosystemic encephalopathy [126], with phenylketonuria [127,128], and patients with severe traumatic brain injury undergoing rehabilitation [129] or in a minimally conscious state following traumatic brain injury [130]. Neurocognitive retrieval after BCAA supplementation was also found in an animal model of traumatic brain injury [131]. Cognitive performance was improved after BCAA supplementation in healthy exercising subjects [132,133]. In a large epidemiological study, low plasma BCAA levels were found to be independent predictors of incident dementia [134].

We postulate that reduced brain BCAA availability may accelerate the progression of AD for at least five main reasons. First, low brain BCAA might impair the turnover of glutamate, the main excitatory neurotransmitter in the brain [135,136] as nitrogen from BCAA is used for the formation of glutamate in astroglia [137,138]. The nitrogen from BCAA is particularly important, as the content of aspartate, the other nitrogen donor, is limited in astroglia [139]. 

Second, low brain BCAA levels may increase neurotoxicity and neurodegeneration by limiting the glutamate–glutamine cycle, leading to accumulations of both glutamate and ammonia [125]. Third, BCAA modulate the balance between excitatory and inhibitory activities by regulating the pools of glutamate, glutamine, and GABA [140]. Fourth, low brain BCAA may reduce protein synthesis for brain tissue repair, sprouting, and circuitry remodeling [141]. Lastly, low BCAA, in particular leucine, may limit the recovery of cognition by reducing insulin production [120].

Of note, in CTRL subjects, the BCAA/EAA CSF/plasma ratio was >1, supporting, under normal conditions, the importance for the brain of maintaining high levels of BCAA among the EAAs. The decreased CSF levels of EAAs lysine and threonine, despite their normal plasma concentrations, may be secondary to increased brain oxidation and/or increased incorporation into brain proteins. These two amino acids are the most essential among EAAs, since their oxidation is irreversible, as they do not undergo transamination activities [142,143]. Consequently, low brain availability of lysine and threonine creates increased risk for brain synthesis activities. Moreover, lysine has potent antiproteolytic activity [144] even under conditions of a low protein diet [145], and indirectly modulates GABAergic transmission by means of its catabolic product, pipecolic acid [113].

Neural plasticity is regulated by protein phosphorylation occurring in serine, threonine, and tyrosine residues (protein serine–threonine kinase) [146]. The neuronal proteins regulated by protein serine–threonine kinase include enzymes for neurotransmitter biosynthesis and degradation, neurotransmitter receptors and transporters, ion channels, and proteins involved in neuronal growth and motility, to name only a few. These physiological serine–threonine kinase activities lead us to suspect that a lack of brain threonine and serine may have a very negative impact on residual brain function in AD.

Reduced alanine may be due to an altered glycolysis pathway, leading to decreased availability of pyruvate to be converted into alanine via the transamination process.

Altered brain methionine metabolism may explain the reduced CSF levels of methionine found in the current study and may also explain the increased homocysteine (a by-product of methionine) levels in AD serum and brains [147] and the association of elevated serum homocysteine levels with a decreased methionine cycle in AD [95].

As, in the current study, plasma methionine could not be detected, we cannot exclude a reduced contribution of plasma methionine to decreased CSF levels. Another mechanism leading to reduced CSF methionine may be its incorporation into proteins, since it is essential for the initiation of protein and nucleic acid synthesis and its active form, S-AdenosylMethionine (SAME), is the most important methyl donor in the body. Methylation processes via SAME stabilize and protect proteins, including myelin, and are involved in the formation of creatine, carnitine, melatonine, and polyamines and in the metabolism of serotonin. Another metabolic condition leading to low methionine may be excessive amino acid conversion to cysteine (and taurine), an important precursor of the antioxidant glutathione, which may undergo a chronic decrease in AD due to high brain ROS formation [148].

Table 14 shows some potential negative effects on residual brain activities and functions in the study ADs resulting from reduced brain AA availability.

### 4.4. Were the Brains of AD Patients Undernourished? Suggestions for Clinical Practice

Under physiologic conditions, the brains of normal adults would seem to be spared from malnutrition because of very low cell proliferation and cell replacement in the brain [149], low cellular protein turnover [150], and the supply of amino acids to the brain from extracerebral tissues, such as skeletal muscle and liver [151].

However, the findings from more recent studies seem to question the assumption that the brain could be protected from malnutrition. First, the brain protein synthesis rate in normal adults was shown to be higher than that of skeletal muscle tissue [152]: brain protein synthesis was 0.17 ± 0.01%/h in the neocortex and 0.13 ± 0.01%/h in the hippocampus, but only 0.05 ± 0.01%/h in skeletal muscle.

The high rates of brain protein synthesis were documented in other previous studies [153,154]. Second, under protein deficiency conditions, the brain utilizes circulating amino acids from protein breakdown occurring in extracerebral body areas, mainly the skeletal muscle [155]. Of interest, sarcopenia in AD patients has been reported to be associated with brain atrophy and altered cognitive performance [55] and, in elderly subjects, with cognitive impairment. Third, the current study is highly suggestive of a lack of EAAs (tryptophan, phenylalanine) in both normonurished ADs and nutritionally deteriorated ones. In addition, the lack of NON EAAs, mainly in nutritionally deteriorated ADs, contributes to reducing the availability of nitrogen for protein synthesis [156].

Thus, we postulate that the brain of ADs, in particular ADs with MNA < 24, might be considered an “undernourished brain”, caused by an unbalanced ratio of brain AA utilization to AA supply from plasma. Theoretically, brain AA overconsumption could lead or contribute over time to impairment of circulating EAAs, particularly in patients with poor or marginal EAA intakes and in sarcopenic patients.

A problem arising from the current study is whether it is possible to reconcile two conflicting factors: the brain’s needs for EAAs/AAs to sustain metabolic processes such as repairing, circuit remodeling, and growth of the brain’s residual functioning areas and, at the same time, the need to avoid enhanced EAA utilization, which results in the synthesis of AD neuropathology markers. It has been hypothesized that a nutritional strategy could involve providing patients with a diet low in protein but with high levels of BCAAs, aromatic amino acids, glutamine, histidine, and threonine [157,158]. Another hypothesized nutritional treatment could be omitting leucine but augmenting the supply of valine and isoleucine [31], which are less potent stimulators of mTOR than leucine [120]. An additional reported nutritional treatment could be a reduction in methionine intake, methionine being responsible for the increase in Aβ and phosphorylated tau levels in the brain [157]. In mouse AD models, methionine restriction was shown to be neuroprotective by diminishing the levels of Aβ formed [159].

Until investigations address these issues, a reasonable approach in clinical practice for patients with AD at present might be the following:

1. Routine determinations of AA levels, both in plasma and CSF, in ADs.
2. Quantification of dietary protein/EAA intakes since the first diagnosis and frequent monitoring over time.
3. Improving physical activity and exercise, aiming at the preservation of/increase in skeletal muscle mass, strength, and function, since muscle tissue is the main body store of AAs.
4. In nutritionally deteriorated patients (MNA < 24 scores), who represented more than half of the ADs considered in this study, a supplementation of EAAs to restore their plasma levels may be needed as EAAs are also vital for maintenance of the structures and functions of extracerebral organs and tissues. Of note, supplementation of the diet with L-serine prevents synaptic loss and behavioral deficits in AD mice [92]. Future studies will address the question of whether, in addition to EAA supplementation, supplying lactate, pyruvate, and ketone bodies may reduce the brain’s need for AAs to generate energy.
5. All these clinical suggestions should be added to an antinflammatory and antioxidant diet [160], along with nutritional interventions aimed at reducing abnormalities in gut microbiota and slowing down the progression of the disease [161].

A well-planned investigation could examine whether, in ADs with normal nutrition (MNA > 24), the prevention of nutritional deterioration and correction of brain tryptophan and phenylalanine levels should be pursued. In these patients, supplementing these two aromatic amino acids might prove useful for improving their content in the brain.

### 4.5. Limitations of Study

The study has several limitations that require further research to be resolved.

A limitation of the present investigation is the small number of subjects studied, particularly controls.

Urea levels were not determined, either in AD or CTRL CSF. Elevated CSF urea in AD would have corroborated the existence of excessive brain AA consumption.

Patients’ intakes of dietary proteins were not quantified. Information regarding amount and type of ingested amino acids, in particular EAAs, would have been very useful for understanding the contribution of poor diet to reduced plasma and CSF EAAs in patients with nutrition deterioration, as well as to reduced CSF tryptophan and phenylalanine in normonurished AD.

Analysis of body composition with measurements of skeletal muscle tissue, the most important AA repository in the body, would have strengthened the discussion.

As a strength of the study, it is important to underline that the adoption of a simple nutritional test (MNA) may be useful in clinical practice for suggesting the existence of altered AA contents both in plasma [46] and CSF, in the absence of a direct measurement of EAAs in both areas.

## 5. Conclusions

The study documents that AD patients, compared to CTRL, had lower CSF AA levels and lower CSF/plasma AA ratios. The reductions in CSF AAs were in part linked to the disease per se, being independent of patients’ nutritional status, and in part linked to deterioration in nutrition.

Future well-planned studies are needed to understand whether and how it is possible to safely improve CSF AA levels in advanced AD patients.

## Figures and Tables

**Table 1:** Non-amino acid variables of the entire population with AD.

Variables	AD Patients
**Demographic variables**	
Age (years)	74.41 ± 8.17
Male gender	46%
Disease duration (years)	3.4 ± 3.2
**Anthropometric variables**	
Education (years)	6.57 ± 8.17
Body weight (kg)	63.30 ± 13.24
Height (cm)	160.63 ± 9.97
Body mass index (kg/m^2^)	24.61 ± 4.87
Mid-arm circumference (cm)	26.15 ± 3.29
**Biohumoral variables**	
Glucose (NV 70–115 mg/dL)	88.88 ± 13.78
Insulin (NV 4–24 µU/mL)	14.10 ± 5.72
HOMA-IR (NV < 2.4)	4.44 ± 2.19
Glycosylated hemoglobin (NV 4.8–5.9%)	5.65 ± 0.85
Total cholesterol (NV < 200 mg/dL)	192.94 ± 32.90
HDL cholesterol (NV: M > 55 mg/dL; F > 65 mg/dL)	57.66 ± 14.77
LDL cholesterol (NV < 100 mg/dL)	116.03 ± 26.95
Transferrin (NV 200–360 mg/dL)	227.29 ± 40.90
Iron (NV 59–158 µg/dL)	86.41 ± 25.80
Triglycerides (NV 0–200 mg/dL)	97.42 ± 38.34
Vitamin B12 (NV 191–663 pg/mL)	302.87 ± 114.49
Folate (NV 3.1–17.5 ng/mL)	6.84 ± 3.75
Creatinine (NV: M 0.73–1.18 mg/dL; F 0.55–1.02 mg/dL)	0.86 ± 0.22
Albumin (NV 55.8-66.1%)	57.65 ± 5.02
Total protein (NV 6.2–7.5 g/dL)	6.24 ± 0.41
White blood cell count (NV 4.00–10.00 × 10^3^ /µL)	6.35 ± 1.64
Red blood cell count (NV: M 4.30–5.70 × 10^6^ /µL; F 3.80–5.20 × 106 /µL)	4.29 ± 0.45
Hemoglobin (NV: M 13.2–17.3 g/dL; F 11.7–15.5 g/dL)	13.06 ± 1.28
Erythrosedimentation rate (NV < 15 mm/1st h)	19.90 ± 21.92
**AD biomarker concentrations in CSF**	
tau protein (NV < 404 pg/mL)	488.25 ± 458.38
p-tau (NV < 56.5 pg/mL)	78.48 ± 34.13
β-amyloid (NV > 599 pg/mL)	511.48 ± 379.68
β-amyloid/tau (NV > 1.6)	2.02 ± 3.13
**Neurocognitive tests Mini Mental State Examination** (MMSE < 24 denotes cognitive impairment)	16.28 ± 6.53

AD: Alzheimer Disease; CSF: Cortical Spinal Fluid; MMSE: Mini Mental State Examination; NV: normal value.

**Table 2:** Cerebrospinal fluid (CSF) amino acid levels (µmol/L) in AD patients and in controls (CTRL).

	CTRL	AD Patients	*p* Value *
Aspartic Acid	1.38 ± 0.52	1.46 ± 0.95	0.38
Glutamic Acid	57.10 ± 18.66	57.41 ± 18.01	0.97
Asparagine	15.41 ± 3.46	3.20 ± 2.83 *	<0.0001
Serine	15.50 ± 5.18	8.00 ± 4.39 *	0.0006
Glutamine	617.50 ± 100.74	248.15 ± 116.49 *	<0.0001
Histidine	9.85 ± 2.51	8.24 ± 3.02	0.13
Glycine	9.36 ± 5.01	4.05 ± 2.35 *	<0.0001
Threonine	38.23 ± 8.98	27.26 ± 15.46 *	0.001
Citrulline	1.56 ± 1.30	1.53 ± 0.94	0.82
Alanine	59.02 ± 19.49	37.45 ± 15.92 *	0.0008
Arginine	34.31 ± 11.09	17.84 ± 5.83 *	<0.0001
Tyrosine	13.02 ± 7.10	9.14 ± 3.68 *	0.023
Cysteine	10.36 ± 4.23	8.23 ± 3.17	0.14
Valine	34.24 ± 14.33	19.84 ± 10.74 *	0.0004
Methionine	4.55 ± 1.86	2.45 ± 1.43 *	0.0004
Tryptophan	4.51 ± 1.88	2.06 ± 1.45 *	<0.0001
Phenylalanine	17.04 ± 4.16	10.33 ± 3.75 *	<0.0001
Isoleucine	11.93 ± 3.56	6.63 ± 3.66 *	<0.0001
Leucine	24.95 ± 8.17	14.16 ± 7.02 *	0.0002
Lysine	32.23 ± 11.10	20.53 ± 6.73 *	0.001
Ornithine	5.21 ± 3.70	4.40 ± 4.94	0.20
Total AAs	922.38 ± 307.78	504.71 ± 191.35 *	0.0003
EAAs	184.78 ± 52.85	111.88 ± 46.25 *	<0.0001
BCAAs	63.21 ± 20.25	40.63 ± 21.25 *	0.0005
BCAAs/TAAs	0.08 ± 0.04	0.08 ± 0.03	0.17
EAAs/TAAs	0.22 ± 0.09	0.23 ± 0.06	0.07
BCAAs/EAAs	0.34 ± 0.04	0.36 ± 0.06	0.48
Trp ratio (%)	0.05 ± 0.01	0.04 ± 0.03 *	0.0003

CTRL: control subjects. TAAs: Total Amino Acids; EAAs: Essential Amino Acids; BCAAs: Branched Chain Amino Acids. Trp ratio: Tryptophan/BCAA + Phenyalalnine + Tyrosine. *p*: indicates the degree of statistical significance. * *p* < 0.05.

**Table 3:** Plasma amino acid levels (µmol/L) in AD patients and in controls (CTRL).

	Controls	AD Patients	*p* Value *
Aspartic Acid	7.33 ± 2.40	7.47 ± 3.91	0.53
Glutamic Acid	89.89 ± 46.50	63.56 ± 18.81 *	0.033
Asparagine	38.11 ± 6.74	39.84 ± 5.09	0.59
Serine	90.67 ± 22.63	99.31 ± 22.39	0.27
Glutamine	514.44 ± 136.72	514.09 ± 82.96	1.00
Histidine	47.89 ± 7.49	67.16 ± 11.99 *	0.00019
Glycine	205.22 ± 66.65	226.47 ± 63.13	0.20
Threonine	101.33 ± 17.33	116.59 ± 25.06	0.19
Citrulline	30.11 ± 14.71	35.50 ± 12.10	0.31
Alanine	456.67 ± 113.67	340.22 ± 71.92 *	0.004
Arginine	49.00 ± 16.17	64.75 ± 22.36 *	0.042
Tyrosine	53.44 ± 12.84	51.81 ± 9.83	0.71
Cysteine	na	na	na
Valine	180.13 ± 43.68	203.23 ± 54.19	0.25
Methionine	na	na	na
Tryptophan	40.89 ± 6.45	43.59 ± 8.62	0.26
Phenylalanine	57.67 ± 9.70	51.16 ± 9.54	0.08
Isoleucine	64.44 ± 17.22	54.44 ± 15.34 *	0.058
Leucine	128.67 ± 31.20	104.22 ± 27.79 *	0.025
Lysine	208.22 ± 42.67	196.75 ± 33.61	0.61
Ornithine	87.11 ± 32.15	82.84 ± 23.78	0.73
Total AAs	2839.81 ± 412.48	2366.48 ± 315.21 *	0.004
EAAs	1115.67 ± 175.91	876.98 ± 159.22 *	0.002
BCAAs	373.24 ± 91.60	361.88 ± 96.20	0.74
BCAAs/TAAs	0.13 ± 0.04	0.15 ± 0.03 *	0.036
EAAs/TAAs	0.39 ± 0.03	0.37 ± 0.03	0.057
BCAAs/EAAs	0.34 ± 0.09	0.41 ± 0.04 *	0.001
Trp ratio (%)	0.09 ± 0.02	0.10 ± 0.02	0.39

CTRL: control subjects. TAAs: Total Amino Acids; EAAs: Essential Amino Acids; BCAAs: Branched Chain Amino Acids. Trp ratio: Tryptophan/BCAA + Phenyalalnine + Tyrosine. *p*: indicates the degree of statistical significance; * *p* < 0.05; na: not available.

**Table 4:** Cerebrospinal fluid amino acid levels (µmol/L) in AD patients, stratified by nutritional state, and in controls (CTRL).

Liquor	CTRL	MNA Grp 1	MNA Grp 2	MNA Grp 3	*p* Global	CTRL vs. MNA1 *p* *	CTRL vs. MNA2 *p* *	CTRL vs. MNA3 *p* *
Aspartic Acid	1.38 ± 0.52	0.82 ± 0.11 *	1.44 ± 1.17	1.76 ± 0.85	0.006	0.026	0.93	0.98
Glutamic Acid	57.10 ± 18.66	45.83 ± 15.08	49.61 ± 20.74	68.51 ± 9.16	0.011	0.68	0.85	0.38
Asparagine	15.41 ± 3.46	2.51 ± 1.42 *	3.43 ± 3.90 *	3.33 ± 2.34 *	<0.0001	0.002	0.00021	0.0006
Serine	15.50 ± 5.18	6.02 ± 1.97 *	8.31 ± 5.46 *	8.66 ± 4.19	0.005	0.013	0.043	0.053
Glutamine	617.50 ± 100.74	192.37 ± 106.76 *	261.70 ± 116.83 *	262.44 ± 121.35 *	<0.0001	0.00035	0.001	0.00042
Hystidine	9.85 ± 2.51	8.21 ± 4.14	7.89 ± 3.38	8.55 ± 2.25	0.39			
Glycine	9.36 ± 5.01	3.13 ± 2.27 *	4.42 ± 3.10 *	4.17 ± 1.63 *	0.0007	0.004	0.011	0.007
Threonine	38.23 ± 8.98	21.35 ± 11.25 *	29.41 ± 21.68 *	28.10 ± 10.97	0.008	0.023	0.049	0.11
Citrulline	1.56 ± 1.30	1.03 ± 0.56	1.40 ± 0.94	1.85 ± 1.01	0.18			
Alanine	59.02 ± 19.49	36.33 ± 18.79	33.44 ± 17.63 *	41.07 ± 13.49	0.002	0.13	0.002	0.24
Arginine	34.31 ± 11.09	15.36 ± 6.55 *	17.83 ± 5.21 *	18.91 ± 6.09 *	0.0005	0.007	0.007	0.006
Tyrosine	13.02 ± 7.10	8.13 ± 4.07	9.68 ± 4.93	9.15 ± 2.33	0.12			
Cysteine	10.36 ± 4.23	6.65 ± 2.49	8.25 ± 3.95	8.89 ± 2.68	0.19			
Valine	34.24 ± 14.33	13.99 ± 7.67 *	18.00 ± 11.62 *	23.78 ± 10.22	0.00035	0.002	0.003	0.35
Methionine	4.55 ± 1.86	1.73 ± 0.99 *	2.35 ± 1.66 *	2.84 ± 1.34	0.002	0.006	0.011	0.12
Tryptophan	4.51 ± 1.88	1.44 ± 0.63 *	1.94 ± 1.01 *	2.42 ± 1.89 *	<0.0001	0.0008	0.002	0.005
Phenylalanine	17.04 ± 4.16	9.21 ± 4.82 *	9.82 ± 4.33 *	11.21 ± 2.73 *	0.0006	0.010	0.002	0.042
Isoleucine	11.93 ± 3.56	4.96 ± 2.42 *	5.52 ± 3.36 *	8.21 ± 3.86	<0.0001	0.002	0.00030	0.15
Leucine	24.95 ± 8.17	10.75 ±5.85 *	12.77 ± 6.88 *	16.73 ± 7.07	0.00024	0.002	0.002	0.18
Lysine	32.2 ±11.10	17.04 ±5.46 *	19.37 ± 8.35 *	22.93 ± 5.13	0.001	0.013	0.004	0.48
Ornithine	5.21 ± 3.70	3.89 ± 2.73	5.42 ± 7.92	3.77 ± 1.46	0.49			
Total AAs	922.38 ± 307.78	411.28 ± 187.85 *	513.10 ± 224.29 *	538.16 ± 164.10 *	0.002	0.006	0.017	0.07
EAAs	184.78 ± 52.85	89.47 ± 41.21 *	108.15 ± 62.05 *	124.42 ± 30.13	0.00018	0.001	0.001	0.11
BCAAs	63.21 ± 20.25	29.70 ± 15.79 *	36.29 ± 21.75 *	48.72 ± 20.99	0.00040	0.003	0.003	0.39
BCAAs/TAAs	0.08 ± 0.04	0.07 ± 0.01	0.07 ± 0.02	0.10 ± 0.04	0.17			
EAAs/TAAs	0.22 ± 0.09	0.22 ± 0.02	0.21 ± 0.04	0.24 ± 0.07	0.16			
BCAAs/EAAs	0.34 ± 0.04	0.33 ± 0.05	0.34 ± 0.04	0.38 ± 0.08	0.23			
Trp ratio (%)	0.05 ± 0.01	0.03 ± 0.01	0.03 ± 0.01 *	0.04 ± 0.05 *	0.002	0.10	0.14	0.001

Group 1: malnutrition, Group 2: at risk, Group 3: normal nutrition. CTRL: control subjects. TAAs: Total Amino Acids; EAAs: Essential Amino Acids; BCAAs: Branched Chain Amino Acids. Trp ratio: Tryptophan/BCAA + Phenyalalnine + Tyrosine. *p*: indicates the degree of statistical significance; * *p* < 0.05.

**Table 5:** Differences in the distribution (%) of individual AAs (n = 20) in CSF and plasma (n = 19) in ADs stratified by nutritional state.

	Group 1 Malnourished	Group 2 At Risk	Group 3 Normonourished	Group 1 + 2 Combined
CSF	70	70	35	70
Plasma	0	0	5.2	26.3

**Table 6:** Differences in the types of altered CSF amino acids in relation to nutrition state in AD patients.

	Group 1 Malnourished	Group 2 At risk	Group 3 Normonurished	Group 1 + 2 Combined
CSF	-	Alanine	-	Alanine
	Aspartate	-	-	-
	* Asparagine	* Asparagine	* Asparagine	Asparagine
	* Serine	* Serine	* Serine	Serine
	* Glutamine	* Glutamine	* Glutamine	Glutamine
	* Glycine	* Glycine	* Glycine	-
	Threonine	Threonine	-	Threonine
	* Arginine	* Arginine	* Arginine	Arginine
	Valine	Valine	-	Valine
	Methionine	Methionine	-	Methionine
	* Tryptophane	* Tryptophane	* Tryptophane	Tryptophane
	* Phenylalanine	* Phenylalanine	* Phenylalanine	Phenylalanine
	Isoleucine	Isoleucine	-	Isoleucine
	Leucine	Leucine	-	Leucine
	Lysine	Lysine	-	Lysine
	Total amino acids	Total amino acids	-	Total amino acids
	Essential amino acids	Essential amino acids	-	Essential amino acids
	Brain chain amino acids	Brain chain amino acids	-	Brain chain amino acids
	-	-		Trp/ratio

* Amino Acid alterations shared by the 3 groups of patients independently of nutritional state.

**Table 7:** Plasma amino acid levels (µmol/L) in AD patients, stratified by nutritional state, and in controls (CTRL).

Plasma	CTRL	MNA Grp 1–2	MNA Grp 3	*p* Global	CTRL vs. MNA 1 + 2 *p* *	CTRL vs. MNA 3 *p* *	MNA1 + 2 vs. MNA 3 *p* ^
Aspartic Acid	7.33 ± 2.40	7.44 ± 4.43	7.50 ± 3.46	0.76			
Glutamic Acid	89.89 ± 46.50	60.38 ± 18.28 *	66.75 ± 19.38	0.051	0.044	0.38	0.57
Asparagine	38.11 ± 6.74	38.56 ± 5.16	41.13 ± 4.83	0.41			
Serine	90.67 ± 22.63	99.31 ± 25.00	99.31 ± 20.27	0.53			
Glutamine	514.44 ± 136.72	504.75 ± 79.54	523.44 ± 87.82	0.96			
Histidine	47.89 ± 7.49	64.44 ± 10.78 *	69.88 ± 12.85 *	0.00047	0.010	0.00032	0.60
Glycine	205.22 ± 66.65	238.06 ± 79.17	214.88 ± 41.02	0.38			
Threonine	101.33 ± 17.33	119.06 ± 25.61	114.13 ± 25.09	0.31			
Citrulline	30.11 ± 14.71	33.25 ± 10.13	37.75 ± 13.76	0.49			
Alanine	456.67 ± 113.67	345.13 ± 84.88 *	335.31 ± 58.61 *	0.016	0.026	0.030	1.00
Arginine	49.00 ± 16.17	63.94 ± 19.13	65.56 ± 25.80	0.12			
Tyrosine	53.44 ± 12.84	54.50 ± 9.61	49.13 ± 9.58	0.38			
Cysteine	na	na	na	na			
Valine	180.13 ± 43.68	181.72 ± 49.84	224.74 ± 50.97 *	0.025	1.00	0.10	0.042
Methionine	na	na	na	na			
Tryptophan	40.89 ± 6.45	42.38 ± 10.42	44.81 ± 6.48	0.34			
Phenylalanine	57.67 ± 9.70	51.44 ± 9.04	50.88 ± 10.31	0.15			
Isoleucine	64.44 ± 17.22	48.88 ± 12.40 *	60.00 ± 16.32	0.044	0.047	0.65	0.28
Leucine	128.67 ± 31.20	93.19 ± 25.56 *	115.25 ± 26.14 *	0.006	0.008	0.62	0.07
Lysine	208.22 ± 42.67	190.88 ± 38.90	202.63 ± 27.35	0.54			
Ornithine	87.11 ± 32.15	81.56 ± 24.84	84.13 ±23.41	0.93			
Total AA	2839.81 ± 412.48	2322.02 ± 363.07 *	2410.94 ± 263.25	0.010	0.008	0.062	0.80
EAAs	1115.67 ± 175.91	830.53 ± 162.84 *	923.42 ± 145.85	0.002	0.002	0.08	0.36
BCAAs	373.24 ± 91.60	323.78 ± 86.86	399.99 ±92.11	0.060			
BCAAs/TAAs	0.13 ± 0.04	0.14 ± 0.02	0.16 ± 0.03 *^	0.004	0.81	0.008	0.027
EAAs/TAAs	0.39 ± 0.03	0.36 ± 0.03 *	0.38 ± 0.03	0.017	0.025	0.78	0.10
BCAAs/EAAs	0.34 ± 0.09	0.39 ± 0.04	0.43 ± 0.04 *^	0.00027	0.15	0.00021	0.050
Trp ratio (%)	0.09 ± 0.02	0.10 ± 0.02	0.09 ± 0.01	0.28			

Group 1–2: malnutrition + at risk, Group 3: normal nutrition. CTRL: control subjects. TAAs: Total Amino Acids; EAAs: Essential Amino Acids; BCAAs: Branched Chain Amino Acids. Trp ratio: Tryptophan/BCAA + Phenyalalnine + Tyrosine. *p*: indicates the degree of statistical significance na: not available. * *p* < 0.05 CTRL vs. MNA1 + 2; * *p* < 0.05 CTRL vs. MNA3; ^ *p* < 0.05 MNA1 + 2 vs. MNA3.

**Table 8:** Cerebrospinal fluid amino acid levels (µmol/l) in AD patients, stratified by nutritional state, and in controls (CTRL).

Liquor	CTRL	MNA Grp1 + 2	MNA Grp 3	*p* Global	CTRL vs. MNA 1 + 2 *p* *	CTRL vs. MNA 3 *p* *	MNA 1 + 2 vs. MNA 3 *p* ^
Aspartic Acid	1.38 ± 0.52	1.22 ± 0.98	1.76 ± 0.85 ^	0.015	0.12	0.87	0.018
Glutamic Acid	57.10 ± 18.66	48.28 ± 18.53	68.51 ± 9.16 ^	0.004	0.38	0.21	0.003
Asparagine	15.41 ± 3.46	3.11 ± 3.22 *	3.33 ± 2.34 *	<0.0001	<0.0001	0.00029	0.95
Serine	15.50 ± 5.18	7.50 ± 4.60 *	8.66 ± 4.19 *	0.002	0.002	0.027	0.91
Glutamine	617.50 ± 100.74	237.23 ± 115.15 *	262.44 ± 121.35 *	<0.0001	<0.0001	0.00021	0.99
Histidine	9.85 ± 2.51	8.01 ± 3.54	8.55 ± 2.25	0.24			
Glycine	9.36 ± 5.01	3.97 ± 2.83 *	4.17 ± 1.63 *	0.00027	0.00041	0.003	0.98
Threonine	38.23 ± 8.98	26.56 ± 18.68 *	28.10 ± 10.97	0.003	0.003	0.056	0.79
Citrulline	1.56 ± 1.30	1.27 ± 0.83	1.85 ± 1.01	0.14			
Alanine	59.02 ± 19.49	34.46 ± 17.51 *	41.07 ± 13.49	0.0010	0.0006	0.13	0.28
Arginine	34.31 ± 11.09	16.96 ± 5.64 *	18.91 ± 6.09 *	0.00017	0.00027	0.003	0.95
Tyrosine	13.02 ± 7.10	9.14 ± 4.58	9.15 ± 2.33	0.07			
Cysteine	10.36 ± 4.23	7.68 ± 3.51	8.89 ± 2.68	0.11			
Valine	34.24 ± 14.33	16.59 ± 10.33 *	23.78 ± 10.22	0.00012	<0.0001	0.20	0.060
Methionine	4.55 ± 1.86	2.13 ± 1.45 *	2.84 ± 1.34	0.0006	0.00039	0.060	0.41
Tryptophan	4.51 ± 1.88	1.76 ± 0.90 *	2.42 ± 1.89 *	<0.0001	<0.0001	0.002	0.74
Phenylalanine	17.04 ± 4.16	9.61 ± 4.37 *	11.21 ± 2.73 *	0.00018	0.00012	0.021	0.49
Isoleucine	11.93 ± 3.56	5.33 ± 3.00 *	8.21 ± 3.86	<0.0001	<0.0001	0.08	0.061
Leucine	24.95 ± 8.17	12.05 ± 6.43 *	16.73 ± 7.07	<0.0001	<0.0001	0.09	0.11
Lysine	32.23 ± 11.10	18.55 ± 7.36 *	22.93 ± 5.13	0.00045	0.00029	0.28	0.08
Ornithine	5.21 ± 3.70	4.88 ± 6.49	3.77 ± 1.46	0.30			
Total AAs	922.38 ± 307.78	477.16 ± 212.10 *	538.16 ± 164.10 *	0.0008	0.0006	0.038	0.61
EAAs	184.78 ± 52.85	101.56 ± 54.97 *	124.42 ± 30.13	<0.0001	<0.0001	0.058	0.14
BCAAs	63.21 ± 20.25	33.97 ± 19.60 *	48.72 ± 20.99 ^	0.00013	<0.0001	0.22	0.055
BCAAs/TAAs	0.08 ± 0.04	0.07 ± 0.02	0.10 ± 0.04	0.09			
EAAs/TAAs	0.22 ± 0.09	0.21 ± 0.03	0.24 ± 0.07	0.09			
BCAAs/EAAs	0.34 ± 0.04	0.33 ± 0.04	0.38 ± 0.08	0.12			
Trp ratio (%)	0.05 ± 0.01	0.03 ± 0.01 *	0.04 ± 0.05 *	0.0007	0.018	0.0007	0.60

Group 1–2: malnutrition + at risk, Group 3: normal nutrition. CTRL: control subjects. TAAs: Total Amino Acids; EAAs: Essential Amino Acids; BCAAs: Branched Chain Amino Acids. Trp ratio: Tryptophan/BCAA + Phenyalalnine + Tyrosine. *p*: indicates the degree of statistical significance. * *p* < 0.05 CTRL vs. MNA1 + 2; * *p* < 0.05 CTRL vs. MNA3; ^ *p* < 0.05 MNA1 + 2 vs MNA3.

**Table 9:** CSF/plasma AA ratios in CTRL and AD patients.

Variable	CTRL	AD	*p* *
Aspartic Acid	0.17 ± 0.05	0.24 ± 0.16	0.27
Glutamic Acid	0.93 ± 0.81	0.98 ± 0.43	0.27
Asparagine	0.43 ± 0.09	0.08 ± 0.07 *	<0.0001
Serine	0.19 ± 0.05	0.08 ± 0.04 *	0.00022
Glutamine	1.34 ± 0.36	0.50 ± 0.27 *	<0.0001
Histidine	0.21 ± 0.05	0.13 ± 0.06 *	0.002
Glycine	0.04 ± 0.02	0.02 ± 0.01 *	<0.0001
Threonine	0.42 ± 0.11	0.23 ± 0.11 *	0.0006
Citrulline	0.05 ± 0.03	0.05 ± 0.03	0.80
Alanine	0.16 ± 0.05	0.11 ± 0.04 *	0.015
Arginine	0.76 ± 0.28	0.30 ± 0.14 *	<0.0001
Tyrosine	0.25 ± 0.08	0.17 ± 0.06 *	0.029
Cysteine	na	na	na
Valine	0.19 ± 0.06	0.10 ± 0.04 *	0.00011
Methionine	na	na	na
Tryptophan	0.10 ± 0.03	0.05 ± 0.03 *	0.00023
Phenylalanine	0.32 ± 0.08	0.20 ± 0.08 *	0.001
Isoleucine	0.22 ± 0.11	0.12 ± 0.05 *	0.001
Leucine	0.22 ± 0.09	0.14 ± 0.06 *	0.003
Lysine	0.18 ± 0.07	0.11 ± 0.04 *	0.003
Ornithine	0.05 ± 0.05	0.05 ± 0.05	0.88
Total AAs	0.38 ± 0.08	0.22 ± 0.08 *	0.00017
EAAs	0.18 ± 0.05	0.13 ± 0.05 *	0.007
BCAAs	0.18 ± 0.05	0.11 ± 0.05 *	0.0010
BCAAs/TAAs	0.48 ± 0.09	0.54 ± 0.17	0.64
EAAs/TAAs	0.48 ± 0.05	0.61 ± 0.14 *	0.002
BCAAs/EAAs	1.02 ± 0.18	0.87 ± 0.11 *	0.006
Trp ratio (%)	0.51 ± 0.16	0.40 ± 0.36 *	0.008

CTRL: control subjects. TAAs: Total Amino Acids; EAAs: Essential Amino Acids; BCAAs: Branched Chain Amino Acids. Trp ratio: Tryptophan/BCAA + Phenyalalnine + Tyrosine. *p*: indicates the degree of statistical significance; * *p* < 0.05; na: not available.

**Table 10:** CSF/plasma AA ratios in relation to patient nutritional state.

Variable	CTRL	MNA Grp 1	MNA Grp 2	MNA Grp 3	*p* Global	CTRL vs. MNA 1 *p* *	CTRL vs. MNA 2 *p* *	CTRL vs. MNA 3 *p* *
Aspartic Acid	0.17 ± 0.05	0.14 ± 0.08	0.23 ± 0.20	0.28 ± 0.13	0.055	0.99	1.00	0.24
Glutamic Acid	0.93 ± 0.81	0.80 ± 0.41	0.88 ± 0.40	1.13 ± 0.43	0.21			
Asparagine	0.43 ± 0.09	0.06 ± 0.03 *	0.09 ± 0.10 *	0.08 ± 0.06 *	0.00042	0.004	0.002	0.002
Serine	0.19 ± 0.05	0.06 ± 0.03 *	0.09 ± 0.05 *	0.09 ± 0.03 *	0.002	0.006	0.013	0.015
Glutamine	1.34 ± 0.36	0.32 ± 0.13 *	0.54 ± 0.28 *	0.54 ± 0.28 *	0.0005	0.002	0.005	0.005
Histidine	0.21 ± 0.05	0.11 ± 0.06	0.14 ± 0.08 *	0.13 ± 0.04 *	0.015	0.052	0.041	0.047
Glycine	0.04 ± 0.02	0.01 ± 0.01 *	0.02 ± 0.01 *	0.02 ± 0.01 *	0.0010	0.005	0.005	0.007
Threonine	0.42 ± 0.11	0.16 ± 0.08 *	0.24 ± 0.13 *	0.25 ± 0.09 *	0.003	0.004	0.028	0.038
Citrulline	0.05 ± 0.03	0.03 ± 0.01	0.04 ± 0.02	0.06 ± 0.04	0.18			
Alanine	0.16 ± 0.05	0.09 ± 0.04	0.10 ± 0.04 *	0.13 ± 0.04	0.019	0.11	0.030	0.72
Arginine	0.76 ± 0.28	0.22 ± 0.12 *	0.30 ± 0.09 *	0.33 ± 0.17 *	0.00043	0.002	0.003	0.004
Tyrosine	0.25 ± 0.08	0.13 ± 0.05 *	0.18 ± 0.08	0.18 ± 0.04	0.043	0.028	0.47	0.54
Cysteine	na	na	na	na	na			
Valine	0.19 ± 0.06	0.07 ± 0.03 *	0.10 ± 0.06 *	0.11 ± 0.03 *	0.0007	0.001	0.007	0.013
Methionine	na	na	na	na	na			
Tryptophan	0.10 ± 0.03	0.03 ± 0.01 *	0.05 ± 0.03 *	0.05 ± 0.04 *	0.002	0.004	0.017	0.012
Phenylalanine	0.32 ± 0.08	0.14 ± 0.06 *	0.21 ± 0.09 *	0.22 ± 0.06	0.003	0.002	0.042	0.13
Isoleucine	0.22 ± 0.11	0.09 ± 0.04 *	0.12 ± 0.07 *	0.14 ± 0.04	0.002	0.005	0.010	0.21
Leucine	0.22 ± 0.09	0.10 ± 0.05 *	0.14 ± 0.07	0.14 ± 0.04	0.017	0.026	0.064	0.12
Lysine	0.18 ± 0.07	0.09 ± 0.04 *	0.11 ± 0.05 *	0.11 ± 0.02	0.017	0.028	0.050	0.14
Ornithine	0.05 ± 0.05	0.04 ± 0.03	0.06 ± 0.07	0.05 ± 0.02	0.98			
Total AAs	0.38 ± 0.08	0.16 ± 0.07 *	0.23 ± 0.10 *	0.23 ± 0.08 *	0.001	0.003	0.017	0.009
EAAs	0.18 ± 0.05	0.10 ± 0.05 *	0.13 ± 0.07	0.14 ± 0.03	0.023	0.024	0.11	0.25
BCAAs	0.18 ± 0.05	0.08 ± 0.04 *	0.12 ± 0.06 *	0.12 ± 0.04	0.005	0.007	0.024	0.08
BCAAs/TAAs	0.48 ± 0.09	0.50 ± 0.06	0.53 ± 0.14	0.57 ± 0.22	0.85			
EAAs/TAAs	0.48 ± 0.05	0.60 ± 0.07	0.60 ± 0.11	0.63 ± 0.18 *	0.023	0.13	0.070	0.030
BCAAs/EAAs	1.02 ± 0.18	0.83 ± 0.03	0.88 ± 0.10	0.89 ± 0.14	0.044	0.11	0.09	0.15
Trp ratio (%)	0.51 ± 0.16	0.35 ± 0.09	0.35 ± 0.13	0.45 ± 0.52 *	0.059	0.45	0.23	0.046

Group 1: malnutrition, Group 2: at risk, Group 3: normal nutrition. CTRL: control subjects. TAAs: Total Amino Acids; EAAs: Essential Amino Acids; BCAAs: Branched Chain Amino Acids. Trp ratio: Tryptophan/BCAA + Phenyalalnine + Tyrosine. *p*: indicates the degree of statistical significance; * *p* < 0.05 na: not available.

**Table 11:** Association between plasma insulin levels, CSF AA levels, and markers of AD pathology.

Variable	Spearman r	Pearson r
Aspartic Acid	0.19 (0.33)	0.07 (0.73)
Glutamic Acid	0.20 (0.30)	0.09 (0.63)
Asparagine	−0.16 (0.41)	−0.16 (0.42)
Serine	−0.24 (0.23)	−0.19 (0.33)
Glutamine	−0.26 (0.19)	−0.24 (0.22)
Histidine	−0.28 (0.15)	−0.31 (0.11)
Glycine	−0.19 (0.33)	−0.26 (0.18)
Threonine	−0.14 (0.45)	−0.22 (0.26)
Citrulline	−0.15 (0.44)	−0.11 (0.58)
Alanine	−0.11 (0.56)	−0.15 (0.45)
Arginine	−0.40 (0.031)	−0.52 (0.004)
Tyrosine	−0.02 (0.93)	−0.22 (0.24)
Cysteine	−0.17 (0.37)	−0.22 (0.25)
Valine	0.09 (0.65)	−0.05 (0.79)
Methionine	0.02 (0.93)	−0.01 (0.96)
Tryptophan	−0.08 (0.67)	0.25 (0.19)
Phenylalanine	−0.04 (0.83)	−0.17 (0.38)
Isoleucine	0.09 (0.64)	−0.01 (0.94)
Leucine	0.03 (0.87)	−0.08 (0.67)
Lysine	−0.07 (0.71)	−0.22 (0.25)
Ornithine	−0.24 (0.22)	−0.21 (0.28)
Total AAs	−0.21 (0.27)	−0.27 (0.16)
EAAs	−0.00 (1.00)	−0.17 (0.37)
BCAAs	0.07 (0.73)	−0.06 (0.78)
BCAAs/TAAs	0.32 (0.09)	0.28 (0.14)
EAAs/TAAs	0.22 (0.25)	0.24 (0.21)
BCAAs/EAAs	0.38 (0.045)	0.26 (0.17)
Trp ratio (%)	−0.33 (0.09)	0.33 (0.08)
tau protein (NV < 404 pg/mL)	0.19 (0.37)	0.00 (1.00)
p-tau (NV < 56.5 pg/mL)	0.22 (0.32)	0.08 (0.72)
β-amyloid (NV > 599 pg/mL)	0.28 (0.18)	0.11 (0.62)
β-amyloid/tau (NV > 1.6)	0.05 (0.83)	0.06 (0.76)

TAAs: Total Amino Acids; EAAs: Essential Amino Acids; BCAAs: Branched Chain Amino Acids. Trp ratio: Tryptophan/BCAA + Phenyalalnine + Tyrosine. p: levels of significance, into the brackets.

**Table 12:** Association between CSF AAs and plasma AAs in AD.

Variable	Spearman r	Pearson r
Aspartic Acid	0.07 (0.73)	0.00 (0.99)
Glutamic Acid	0.18 (0.34)	0.13 (0.50)
Asparagine	0.09 (0.65)	0.10 (0.61)
Serine	0.12 (0.53)	0.22 (0.26)
Glutamine	−0.37 (0.049)	−0.31 (0.10)
Histidine	−0.30 (0.11)	−0.33 (0.08)
Glycine	0.13 (0.49)	0.18 (0.34)
Threonine	0.14 (0.46)	0.29 (0.13)
Citrulline	0.09 (0.62)	0.27 (0.16)
Alanine	0.25 (0.18)	0.21 (0.27)
Arginine	0.14 (0.46)	0.21 (0.27)
Tyrosine	0.21 (0.26)	0.24 (0.20)
Cysteine	na	na
Valine	0.35 (0.055)	0.37 (0.044)
Methionine	na	na
Tryptophan	−0.07 (0.72)	0.02 (0.92)
Phenylalanine	−0.05 (0.81)	−0.04 (0.82)
Isoleucine	0.34 (0.067)	0.46 (0.011)
Leucine	0.32 (0.09)	0.40 (0.027)
Lysine	0.10 (0.59)	0.04 (0.83)
Ornithine	−0.18 (0.36)	0.18 (0.36)
Total AAs	−0.12 (0.53)	−0.12 (0.51)
EAAs	0.17 (0.36)	0.10 (0.59)
BCAAs	0.36 (0.054)	0.40 (0.030)
BCAAs/TAAs	0.49 (0.006)	0.56 (0.001)
EAAs/TAAs	0.40 (0.028)	0.37 (0.047)
BCAAs/EAAs	0.58 (0.001)	0.72 (<0.0001)
Trp ratio (%)	0.03 (0.90)	−0.01 (0.95)

TAAs: Total Amino Acids; EAAs: Essential Amino Acids; BCAAs: Branched Chain Amino Acids. Trp ratio: Tryptophan/BCAA + Phenyalalnine + Tyrosine. p: levels of significance, into the brackets; na: not available.

**Table 13:** Association between CSF AAs and plasma AAs in controls.

Variable	Spearman r	Pearson r
Aspartic Acid	−0.01 (0.99)	−0.13 (0.76)
Glutamic Acid	−0.23 (0.59)	−0.23 (0.59)
Asparagine	0.45 (0.27)	0.36 (0.39)
Serine	0.96 (0.0007)	0.80 (0.018)
Glutamine	0.13 (0.76)	0.41 (0.32)
Histidine	−0.24 (0.58)	−0.19 (0.65)
Glycine	0.14 (0.75)	0.56 (0.15)
Threonine	0.17 (0.70)	0.14 (0.75)
Citrulline	0.31 (0.46)	0.30 (0.47)
Alanine	−0.21 (0.62)	−0.00 (0.99)
Arginine	0.57 (0.15)	0.62 (0.10)
Tyrosine	0.00 (1.00)	0.15 (0.72)
Cysteine	na	na
Valine	0.74 (0.046)	0.61 (0.10)
Methionine	na	na
Tryptophan	−0.10 (0.84)	0.00 (1.00)
Phenylalanine	−0.29 (0.50)	−0.08 (0.86)
Isoleucine	0.17 (0.70)	0.01 (0.98)
Leucine	0.10 (0.84)	0.24 (0.57)
Lysine	−0.24 (0.58)	0.07 (0.88)
Ornithine	0.14 (0.75)	0.06 (0.90)
Total AA	0.29 (0.50)	0.24 (0.56)
EAAs	−0.05 (0.93)	0.08 (0.85)
BCAAs	0.69 (0.069)	0.48 (0.23)
BCAAs/TAAs	0.90 (0.005)	0.50 (0.21)
EAAs/TAAs	0.38 (0.36)	0.51 (0.20)
BCAAs/EAAs	0.60 (0.13)	0.32 (0.44)
Trp ratio (%)	0.62 (0.11)	0.53 (0.18)

TAAs: Total Amino Acids; EAAs: Essential Amino Acids; BCAAs: Branched Chain Amino Acids. Trp ratio: Tryptophan/BCAA + Phenyalalnine + Tyrosine. p: indicates the degree of statistical significance, na: not available.

**Table 14:** Some potential impacts on brain metabolic activities due to reduced brain AAs.

Reduced CSF AAs	Effects on Brain Metabolism	Effects on Brain Activities
Serine	Reduced NMDA glutamate receptor	Reduced formation of new synapses
	Reduced protein–serine kinase	Reduced enzyme synthesis of neurotransmitter biosynthesis and degradation
Glycine	Reduced activity inhibition	Contribution to synaptic and brain toxicity
Asparagine	Reduced stimulation of ornithine decarboxylase	Reduced urea cycle activity
Glutamine	Reduced astrocyte detoxification of ammonia Aβ reduced autophagy Difficulties in maintaining synaptic transmission	Hyperammonia Maintenance of altered mitochondria Contribution to reducing synaptic transmission
Arginine	Disruption of urea cycle Elevated production of NO, nitrosative stress	Hyperammonia Mitochondrial dysfunction Degeneration of synapses and neurons Endothelial dysfunction Reduced synthesis of creatine Increased energy deficit
Tryptophan	Reduced serotonin formation	Reduced serotoninergic neurotransmission Contribution to AD pathogenesis, severity, cognitive impairments
Phenylalanine	Reduced synthesis of dopamine, norepinephrine, epinephrine, tyramine	Reduced catecholaminergic neurotransmission Impairments in behavioural and cognitive functioning Increased perception of fatigue
BCAAs	Impairment in glutamate turnover Impairment in regulation of glutamate, glutamine, GABA Reduced protein synthesis	Increased neurotoxicity Unbalanced excitatory/inhibitory ratio Reduced processes of repairing, sprouting, circuity remodelling
Lysine	Reduced protein synthesis Reduced autoproteolytic activity Reduced formation of pipecolic acid	Increased catabolic activity Reduced modulation of GABAergic transmission
Threonine	Reduced protein synthesis Reduced protein threonine kinase	Increased catabolic activity Reduced synthesis of enzymes for neurotransmitter biosynthesis and degradation, neurotransmitter receptors and transporters, ion channels Reduced neural plasticity
Methionine	Reduced methyl production (methylation)	Reduced initiation of protein synthesis Reduced protein protection (e.g. myelin) Reduced formation of creatine, carnitine, melatonin, and polyamines, reduced metabolism of serotonin Reduced formation of cysteine and hence glutathione formation
Alanine	Reduced transamination process	Reduced energy generation

BCAAs = Branched Chain Amino Acids.

## Data Availability

Data available on request due to restrictions, e.g., privacy or ethical. The data presented in this study are available on request from the corresponding author. The data are not publicly available due to the rules of Mondino Foundation but might be available only on request.
